# *Salix* spp. Bark Hot Water Extracts Show Antiviral, Antibacterial, and Antioxidant Activities—The Bioactive Properties of 16 Clones

**DOI:** 10.3389/fbioe.2021.797939

**Published:** 2021-12-16

**Authors:** Jenni Tienaho, Dhanik Reshamwala, Tytti Sarjala, Petri Kilpeläinen, Jaana Liimatainen, Jinze Dou, Anneli Viherä-Aarnio, Riikka Linnakoski, Varpu Marjomäki, Tuula Jyske

**Affiliations:** ^1^ Production Systems, Natural Resources Institute Finland (Luke), Helsinki, Finland; ^2^ Department of Biological and Environmental Science, Nanoscience Center, University of Jyväskylä, Jyväskylä, Finland; ^3^ Department of Bioproducts and Biosystems, School of Chemical Engineering, Aalto University, Espoo, Finland; ^4^ Natural Resources, Natural Resources Institute Finland (Luke), Helsinki, Finland

**Keywords:** antimicrobial, antioxidant, antiviral, bark, debarking, *Salix* spp., water-extracts

## Abstract

Earlier studies have shown that the bark of *Salix* L. species (Salicaceae family) is rich in extractives, such as diverse bioactive phenolic compounds. However, we lack knowledge on the bioactive properties of the bark of willow species and clones adapted to the harsh climate conditions of the cool temperate zone. Therefore, the present study aimed to obtain information on the functional profiles of northern willow clones for the use of value-added bioactive solutions. Of the 16 willow clones studied here, 12 were examples of widely distributed native Finnish willow species, including dark-leaved willow (*S. myrsinifolia* Salisb.) and tea-leaved willow (*S. phylicifolia* L.) (3 + 4 clones, respectively) and their natural and artificial hybrids (3 + 2 clones, respectively). The four remaining clones were commercial willow varieties from the Swedish willow breeding program. Hot water extraction of bark under mild conditions was carried out. Bioactivity assays were used to screen antiviral, antibacterial, antifungal, yeasticidal, and antioxidant activities, as well as the total phenolic content of the extracts. Additionally, we introduce a fast and less labor-intensive steam-debarking method for *Salix* spp. feedstocks. Clonal variation was observed in the antioxidant properties of the bark extracts of the 16 *Salix* spp. clones. High antiviral activity against a non-enveloped enterovirus, coxsackievirus A9, was found, with no marked differences in efficacy between the native clones. All the clones also showed antibacterial activity against *Staphylococcus aureus* and *Escherichia coli*, whereas no antifungal (*Aspergillus brasiliensis*) or yeasticidal (*Candida albicans*) efficacy was detected. When grouping the clone extract results into *Salix myrsinifolia*, *Salix phylicifolia*, native hybrid, artificial hybrid, and commercial clones, there was a significant difference in the activities between *S. phylicifolia* clone extracts and commercial clone extracts in the favor of *S. phylicifolia* in the antibacterial and antioxidant tests. In some antioxidant tests, *S. phylicifolia* clone extracts were also significantly more active than artificial clone extracts. Additionally, *S. myrsinifolia* clone extracts showed significantly higher activities in some antioxidant tests than commercial clone extracts and artificial clone extracts. Nevertheless, the bark extracts of native Finnish willow clones showed high bioactivity. The obtained knowledge paves the way towards developing high value-added biochemicals and other functional solutions based on willow biorefinery approaches.

## 1 Introduction

Willows (genus *Salix* L.) correspond to approximately 450 species of deciduous trees and shrubs, which are mostly found in moist soils of the Northern Hemisphere (Christenhusz et al., 2017). In general, the major components of willows’ biomass are cellulose, hemicellulose, and lignin, while various minor constituents include flavonoids and other polyphenols (Yan et al., 2021). Willow leaves and bark have long been known as herbal medicines because of their ability to relieve fever and aches. These properties have been attributed to compounds identified from willow species, such as salicinoids and various polyphenols. Salicinoids (syn. salicylates) are phenolic glucosides, which are derivatives of salicyl alcohol, and are commonly found at high levels in the bark and leaves of willows. Most of the salicinoids are signature compounds of *Salix* and *Populus* L. species, and over 20 individual salicinoids have been characterized. Whereas salicylic acid is a ubiquitous plant hormone. Salicin is the simplest and most common salicinoid compound; however, it is often found at low quantities depending on the willow hybrid. Other salicinoids in willows are formed by the esterification of one or more hydroxyl groups of salicin with organic acids, such as benzoic acid in populin and 1-hydroxy-6-oxocyclohex-2-en-1-carboxylic acid in salicortin (Boeckler et al., 2011; Julkunen-Tiitto and Virjamo, 2017). Other small phenolic glycosides common in willow bark are picein, a glucoside of hydroxyacetophenone, salidroside, a glucoside of phenylethanoid, and derivatives of cinnamic alcohols such as triandrin and vimalin (Julkunen-Tiitto, 1985; Kammerer et al., 2005; Dou et al., 2018). Proanthocyanidins up to 20% of bark dry weight (Heiska et al., 2007) and several flavonoids belonging to flavan-3-ols, flavonols, flavanones, and chalcones have also been characterized. A comprehensive review of the phytochemistry and pharmacological activities of *Salix* spp. was recently published by Tawfeek et al. (2021).

**GRAPHICAL ABSTRACT:**
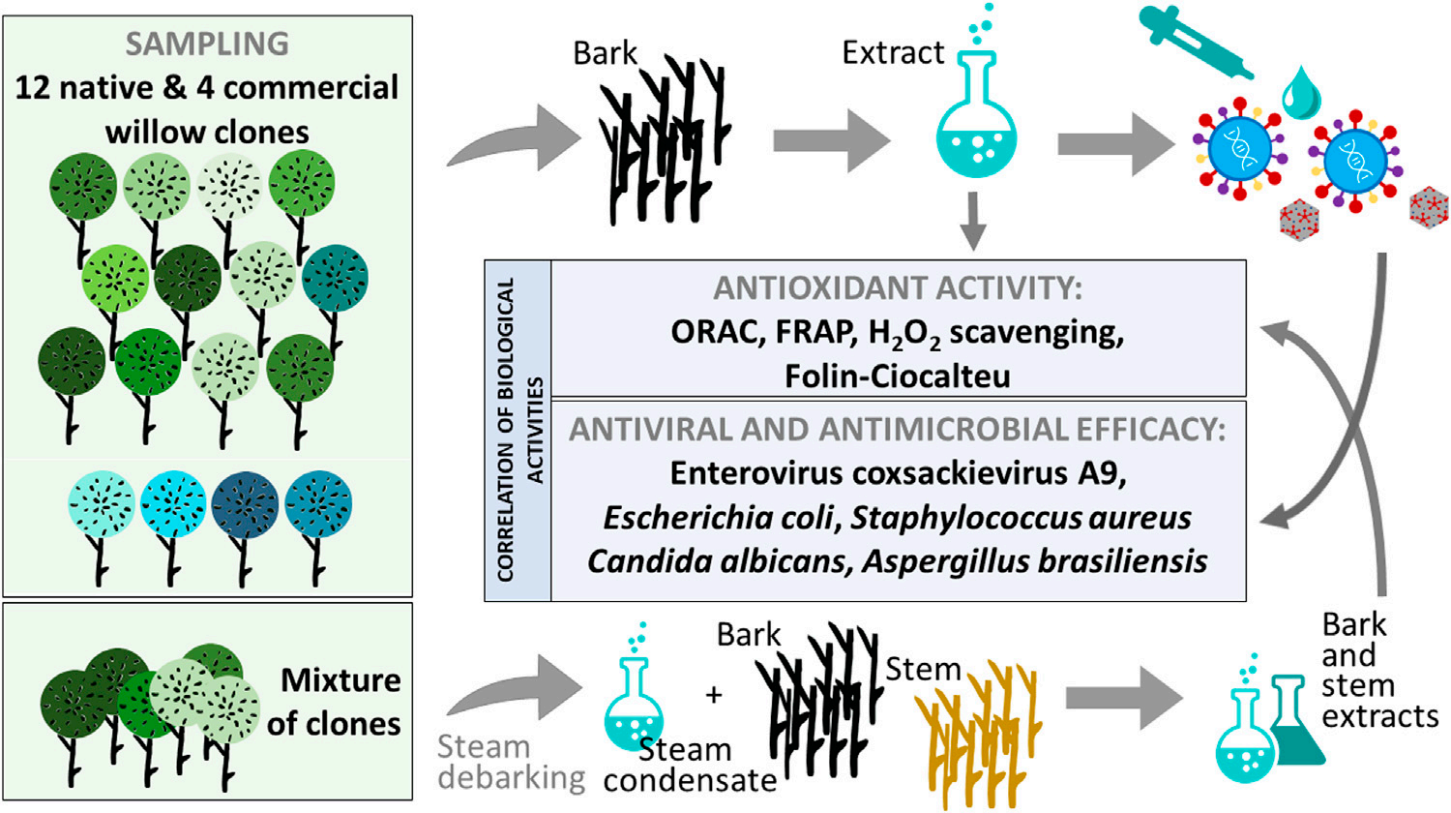


Salicinoids of willow bark can decompose into salicylic acid, which has been found to possess anti-inflammatory and antiviral properties (Singh et al., 2004; Wood, 2015). Highly purified proanthocyanidin fractions of *Salix* spp. extract have also been reported to have antiviral and antibacterial activities (Quosdorf et al., 2017). Overall, proanthocyanidins, or condensed tannins, have been characterized by many biological effects, including antioxidant, antibacterial, antitumor, anticancer, neuroprotective, hypoglycemic, and lipid-lowering activities with a comprehensive positive impact on gastrointestinal health (Yan et al., 2021). Saracila et al. (2018) observed that by feeding the bark extract of *Salix alba* L. to broilers, the number of pathogenic bacteria (Enterobacteriaceae, *Escherichia coli*) on the cecal microbial population decreased while the number of beneficial lactobacilli increased. Three *Salix* spp. bark extracts were also found to have bactericidal effects against *Staphylococcus aureus*, with no significant differences between these species (Ramos et al., 2019). Polyphenols from *Salix tetrasperma* Roxb. stem bark extract were also found to be effective in inhibiting the quorum sensing and virulence of *Pseudomonas aeruginosa* (Mostafa et al., 2020). Malterud et al. (1985) showed that the flavonoid composition from *Salix caprea* L. wood was able to inhibit rot producing wood-destroying fungi *Caniophora puteana*, *Sporotrichum pulverulentum*, and *Trichoderma viride*. Furthermore, willow bark extracts are known to have strong antioxidant and radical scavenging properties (Durak and Gawlik-Dziki, 2014; Bounaama et al., 2016; Ramos et al., 2019). Pharmacological studies have revealed interesting aspects of antitumor and anticancer therapy, including the discovery of a novel cyclodimeric salicinoid, miyabeacin, from *Salix miyabeana* Seemen. and *Salix dasyclados* Wimm. (El-Shemy et al., 2007; Ward et al., 2020). Willow extracts can also be used to relieve pain, inflammation, and fever in a wide variety of conditions with minor adverse effects (Chrubasik et al., 2000; Vlachojannis et al., 2009; Shara and Stohs, 2015). As only mild cytotoxicity has been discovered in willow bark extracts, willows are a promising biomass for various health applications (Ramos et al., 2019). However, because of the vast number of different willow species and their widespread ability to form hybrids, as well as recently identified compounds (e.g., Wu et al., 2016; Noleto-Dias et al., 2018; Noleto-Dias et al. 2019; Noleto-Dias et al. 2020; Ward et al., 2020), there is still much to explore regarding *Salix* spp. and their metabolites.

The biomass of fast-growing willows is recognized as a suitable raw material for biorefineries (Parajuli et al., 2015). Willows can be grown in low-quality agricultural land that cannot be used for food production, thereby reducing competition between food and biomass production (Krzyżaniak et al., 2016). Also, short-rotation woody crop management is less energy consuming than that required for food crops (Djomo et al., 2011). In addition to its potential as a lignocellulosic option for biofuels and bioenergy, willows can be exploited as a renewable source of biochemicals (Brereton et al., 2017). To fully utilize willows’ biomass, both bark and stem wood must be separately valorized (Dou et al., 2016). Carbonized willow bark and wood can be used in supercapacitors (Phiri et al., 2019; Hobisch et al., 2020) and fiber composites (Dou et al., 2019). The cascading use of biomass would be preferential. Isolated biomass fractions should be used as reusable products as much as possible and, finally, after a cycle of reasonable use, compounds and materials should be used as energy after their combustion or anaerobic digestion. For example, the polyphenol containing fraction can be extracted first with hot water, and then the remaining material could be pyrolyzed and anaerobically gasified (Rasi et al., 2019) or used in the production of biochar by slow pyrolysis technology (Rasa et al., 2021). Hot water extraction has been shown to be able to achieve the maximal extract yield from willow bark at 80°C for 20 min (Dou et al., 2018).

Extensive characterization and quantification have been conducted on the components of pharmaceutical preparations from *Salix* spp. (Kammerer et al., 2005) and willow species’ phytochemicals extracted from the bark (Julkunen-Tiitto, 1985; Heiska et al., 2007; Lavola et al., 2018), leaves (Ikonen et al., 2002; Lavola et al., 2018), and whole twigs/biomass (Julkunen-Tiitto, 1985; Brereton et al., 2017). Willow bark is one of the most bioactive compound-rich plant parts (Lavola et al., 2018; Tyśkiewicz et al., 2019), but leaves are also a promising source of polyphenols and antioxidants (Piatczak et al., 2020). However, the content of bark phytochemicals is known to vary among *Salix* spp. (Julkunen-Tiitto, 1985) because of seasonal and environmental factors (Förster et al., 2008), as well as among genotypes and developmental stages of the plant (Lavola et al., 2018). Although the variation of phytochemicals between *Salix* spp. genotypes is mainly quantitative, there can be large differences in compound composition between species and hybrids, which could be a result of the effortless hybridization of *Salix* spp. (Julkunen-Tiitto and Virjamo, 2017). Nevertheless, we lack knowledge on the bark bioactive properties of willow species and/or clones that are well adapted to the northern areas of the cool temperate zone. Therefore, the present study focused on the bark extracts of Finnish willows by screening their antioxidant, antiviral, antibacterial, and antifungal properties.

First, we screened the bioactive properties, i.e., the antioxidant, antiviral (enterovirus strain coxsackievirus A9), antibacterial [*E. coli* (Gram-negative) and *S. aureus* (Gram-positive)], antifungal (*Aspergillus brasiliensis*), and yeasticidal (*Candida albicans*) activities of 16 aqueous bark extracts to the potential of willow materials for various biochemicals and functional products. Additionally, we tested steam-debarking at the 300-L scale as a potentially less-laborious debarking method for northern cultivated willows. We hypothesized that (1) the bark extracts of native willows of local origin have higher biological activity than those of commercial willows due to higher resource allocation to secondary metabolites in native willows; (2) between-species variation in the biological activity of bark extracts offsets the variation among clones; (3) the biological activities of bark extracts are highly intercorrelated; for example, antiviral efficacy can be predicted by the total phenolic content and antibacterial power; (4) individual compounds do not explain the biological activities of crude bark extracts but the extract antioxidant activity and efficacies against viruses and bacteria are due to the synergistic effects of several compounds together—to challenge this pure commercial compounds were also tested; (5) when grouping the clones into *S. phylicifolia*, *S. myrsinifolia*, native hybrid, artificial hybrid, and commercial clones, significant differences can be detected; and (6) when compared to conventional debarking methods, steam-aided debarking is an efficient and less laborious process resulting in *Salix* spp. products with high bioactive potential.

## 2 Materials and Methods

### 2.1 *Salix* spp. Sample Collection

The study materials consisted of 16 willow clones. Of these, 12 clones originated from two common and widely distributed native willow species, dark-leaved willow (*S. myrsinifolia* Salisb.) and tea-leaved willow (*S. phylicifolia* L.) (Väre et al., 2021), as well as from their natural and artificial hybrids. The other four clones were commercial willow varieties from the Swedish willow breeding program (Table 1). Herbarium specimens of the native materials were collected, the identification of the willow species was verified, and the plant specimens were deposited at the Finnish Museum of Natural History, Botanical Museum (H).

**TABLE 1:** Willow clones used for the screening of biological activities.

Sample number	Clone	Species	Type
1	E6682	*S. myrsinifolia*	Native
2	E6771	*S. myrsinifolia*	Native
3	E6948	*S. myrsinifolia*	Native
4	E6666	*S. phylicifolia*	Native
5	K2191	*S. phylicifolia*	Native
6	K2218	*S. phylicifolia*	Native
7	K2277	*S. phylicifolia*	Native
8	K2183	*S. myrsinifolia* × *phylicifolia*	Native hybrid
9	K2269	*S. myrsinifolia* × *phylicifolia*	Native hybrid
10	K2341	*S. myrsinifolia* × *phylicifolia*	Native hybrid
11	V7545	(K2183 *S. myrs.* × *phyl*.) × S15136 *S. gmelinii* ^a^	Artificial hybrid
12	V7546	(K2183 *S. myrs.* × *phyl.)* × P6011 *S. gmelinii* ^a^	Artificial hybrid
13	Scherenee		Commercial clone
14	Tordis		Commercial clone
15	Tora		Commercial clone
16	Klara		Commercial clone

a *Salix gmelinii* Pall. is former *S. dasyclados* Wimm. (Väre et al., 2021).

The native willow clones and artificial hybrids (sample numbers 1–12) were rooted in 20-cm-long cuttings in polystyrene containers (TA913) in a greenhouse at the Haapastensyrjä field station (N60°43′0.01″ E24°26′60.00″), Natural Resources Institute Finland, in the spring of 2017. The growing medium used was a mixture of 2/3 Kekkilä FPM 420 F6 HS *Sphagnum* peat and 1/3 perlite (size 2–6 mm). The containerized plants were moved to the nursery of the Piikkiö field station (N60°25′29.32″ E22°30′57.64″), Natural Resources Institute Finland, in June 2017, where they were grown for the next 2 years. The plants were watered and fertilized according to the normal nursery practices. On May 7, 2019, the 2-year-old willow plants were cut down and the harvested shoots of each clone were packed separately in plastic bags and immediately frozen at –20°C. The harvested willow coppice varied between 1 and 1.5 m in length and 0.5–2 cm diameter at the base.

Commercial willow clones (sample numbers 13–16) were grown by Carbons Finland Ltd. in a peat field at Aitomäki, Kouvola south-eastern Finland (N60°52′0.01″ E26°41′60.00″) from 2016 to March 2019, when the 3-year-old coppice was cut down on March 30 and taken for the study. The 3-m-long sample shoots were cut to shorter, ca. 40-cm-long, pieces and frozen (–20°C) until further treatment.

Two shoots of each willow clone were debarked 50 cm from the base and pooled. The bark was cut into small pieces, frozen at –80°C, and finally freeze-dried. The freeze-dried material was ground with a Moulinex grinder to 1- to 2-mm pieces and kept frozen at –80°C until water extraction.

In addition, *Salix* Klara for the 2-L stirring reactor extraction was provided by Carbons Finland Ltd. from the same growth environment and site, except from spring 2017 to 2020. The willow banks were partly cut down in spring 2018 and 2019 and, for the present study, were cut and gathered on October 24, 2020. The material was then debarked and immediately placed in a freezer (–20°C). The bark was ground with a Kamas cutting mill with a 2-cm sieve.

The material for the pilot-scale bark removal by steaming was collected from a willow clone bank growing at the Piikkiö field station, Finland. The clone bank was established in the summer of 2007. Part of it was cut down in 2013, and all the clones were cut down again on April 4–5, 2019. Material for the steaming experiment was collected as two sample lots, the first containing a mixture of five 6-year-old *Salix purpurea* L. clones. The second sample included a 12-year-old mixture of five *Salix daphnoides* Vill. clones, one *S. purpurea* clone, and two unknown clones. Finally, these two samples were combined, and a bulk sample was used for the steam debarking experiment to ensure the material availability for testing the suitability of the method in general. Detailed information about the samples and their origin can be found in the Supplementary Appendix Table SA1.

### 2.2 *Salix* spp. Clone Extractions

*Salix* spp. samples were freeze-dried before the extraction. Bark was extracted using an ASE-350 accelerated solvent extractor (Dionex, Sunnyvale, CA, United States). The bark sample (1 g) was placed in a stainless-steel extraction vessel (22 ml). The sample was then extracted three times for 15 min with hot water (1:66, w/v) at 90°C, and the extract was stored at –20°C before further analyses.

#### 2.2.1 *Salix* Klara 2-L Stirring Reactor Extractions

Klara bark was extracted using a 2-L stirring reactor (Polyclave, Büchi, Switzerland). The fresh willow bark sample (358 g, corresponding to 150 g dry weight) was placed in the reactor and extracted in a 1:10 bark dry weight/water ratio. The extraction temperature was 80°C, and there was constant stirring (60 rpm) during the 60-min extraction. Solids were separated by collecting liquid through a 50-µm sintered metal filter at the bottom of the reactor. The extract was cooled to room temperature using a heat exchanger. In total, four extractions were carried out under the same conditions, and the average measured total dissolved solids of the extracts was 13.7 ± 0.2 wt% of the dry weight of the original bark. After extraction, the bark extracts were combined into one sample. The extract was concentrated using a rotary evaporator at 60°C, freeze-dried, and the dried extract was ground into powder with a mortar and pestle.

#### 2.2.2 Willow Steam Debarking and Extractions

**FIGURE 1:**
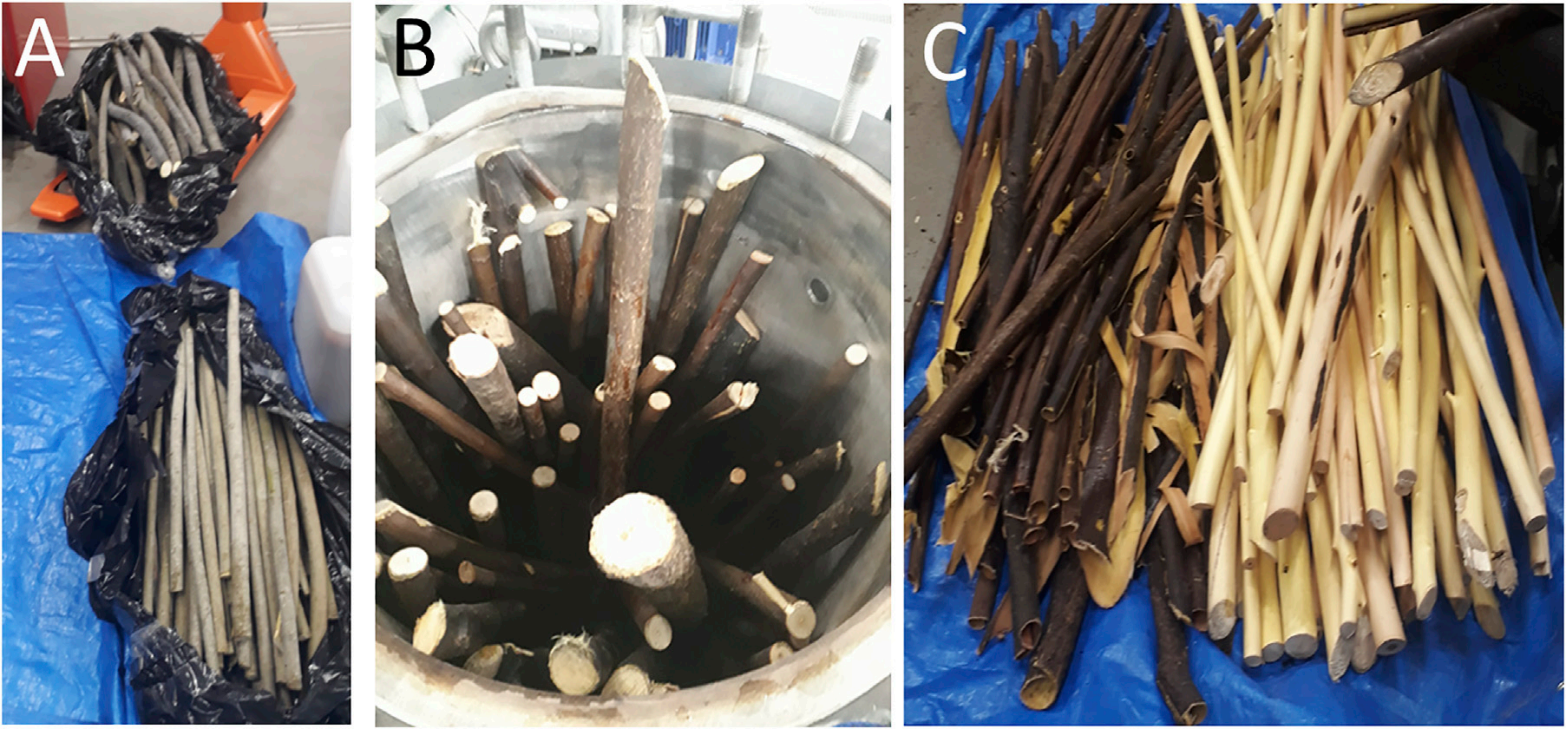
Original willow samples **(A)**, combined willow sticks in reactor **(B)**, and steam-treated bark and woody material **(C)**.

A batch of the combined samples of willow sticks was first steam-treated in a 300-L reactor to remove bark (Figure 1). Samples of steam-treated bark and wood were then extracted at small scale to isolate polyphenol- and carbohydrate-containing fractions. Bark samples were extracted in hot water (90°C) with ASE-350 to isolate polyphenols and wood samples were extracted with a pressurized hot water flow-through extraction system with a 50-ml extraction vessel at 150–180°C to extract carbohydrates. Details of the flow-through extraction system can be found in Kilpeläinen et al. (2014).

Fresh willow sticks (42.2 ± 0.2 kg) were loaded inside the 300-L extraction system that was used in the experiments (Kilpeläinen et al., 2014). For steam collection, 30 L of water was weighed into a tub, and steam was directed to water in the tub with a hose from the extraction system. The temperature inside the vessel was measured during the treatment. Continuous steam flow of 1.6 kg/min (214°C) was passed into the vessel containing willow samples for 20 min. The temperature inside the reactor increased to 100°C after 5–6 min. After 7.5 min, steam started to come out of the vessel, and it was collected into the water-filled tub. When steaming ended after 20 min, the temperature increased to 130°C and the pressure increased to 1.7 bar with a fully open exhaust line.

Steam (3.6 ± 0.2 kg) was collected by the steam collection tub. The condensate was removed *via* the reactor’s drain valve and weighed (19.2 ± 0.2 kg). The steam-treated willow sticks could be easily debarked by hand, as the bark was barely attached to the wood. Samples of the steam condensates, steam-treated bark, and wood were collected and frozen (–20°C) before further analysis.

After the willow steam treatment, the woody material was extracted with a pressurized hot water flow-through system (Kilpeläinen et al., 2012) to isolate fractions with the willow hemicelluloses. Pressurized hot water flow-through extraction has been used to extract hemicelluloses from woody materials (Kilpeläinen et al., 2012; Kilpeläinen et al., 2014). Before extraction, the sample was pre-steamed at 100°C to prevent water channeling through the sample during extraction. Samples (10 g o.d.) were extracted at 150–180°C with a flow rate of 4 ml/min for 60 min. The extract was collected, diluted to 250 ml, and stored in a freezer (–20°C) before analysis.

**FIGURE 2:**
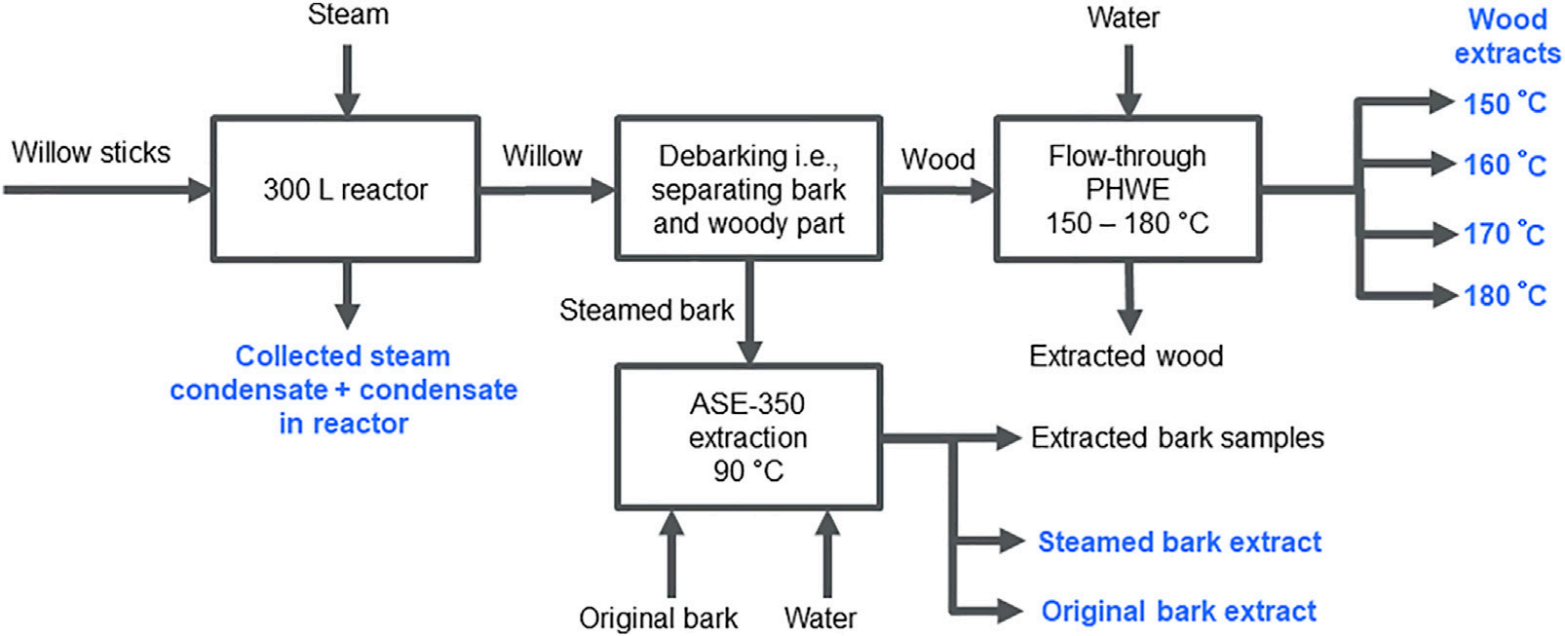
Willow steam treatment, debarking, and extractions. Willow steam treatment condensate, original willow bark ASE-350 extract, and steam-treated bark ASE-350 extract contained polyphenols. Carbohydrates were extracted from woody samples at temperatures from 150 to 180°C after debarking. Collected samples are indicated with blue color.

Steamed bark was extracted with hot water using ASE-350 under the same conditions as the *Salix* spp. clones. Willow samples and treatment techniques used are shown in Figure 2.

### 2.3 Commercial Substances and Samples

Commercial substances were used as references in the antibacterial and antiviral measurements. Salicin and picein (purity >98%) were purchased from Merck Life Science Oy. Salicylic acid (purity >99%) was obtained from VWR Chemicals and triandrin (purity 85%) was obtained from Molport EU. Additionally, Salixin Organic Powder (48^TM^) and Salixin Organic Extract (800NP^TM^) were supplied by Søren Fisker (Salixin A/S) and Pia Wikström (OY CELEGO AB) and were tested for their antibacterial and antiviral efficacy along with the reference substances.

### 2.4 Bioactive Efficacy

#### 2.4.1 Antioxidant Properties

The antioxidant properties of the *Salix* spp. clone bark extracts were tested using the oxygen radical absorbance capacity (ORAC) assay, ferric reducing antioxidant power (FRAP), H_2_O_2_ scavenging test, and Folin-Ciocalteu assay for total phenolic content. All antioxidant assays were carried out using a Varioskan Flash multimode reader (Thermo Scientific) in a 96-well format. The tests covered different antioxidant mechanisms: hydrogen atom transfer (ORAC), single electron transfer (FRAP), and radical scavenging (H_2_O_2_ scavenging). All tests were performed using internal standards with which the sample results were compared.

##### Oxygen Radical Absorbance Capacity

This assay is based on hydrogen atom transfer (HAT) and measures the reduction in fluorescence signal caused by the oxidative dissociation of fluorescein in the presence of peroxyl radicals (R-O-O•) (Huang et al., 2002; Prior et al., 2003). The inhibition of fluorescein breakdown indicates the antioxidant’s protective ability. The experimental setup is described in detail by Tienaho et al. (2020). Briefly, the assay was carried out using four dilutions of each sample, with two technical replicates, by mixing the sample in 0.075 M phosphate buffer pH 7.5 (Merck) with 8.16 × 10^–5^ mM fluorescein (Sigma-Aldrich Chemie GmbH, Steinheim, Germany) and 2,2′-azobis(2-methylpropionamidine) dihydrochloride (Sigma-Aldrich Chemie GmbH, Steinheim, Germany). Trolox (vitamin E analog) ((±)-6-Hydroxy-2,5,7,8-tetramethylchromane-2-carboxylic acid) (Sigma-Aldrich Chemie GmbH, Steinheim, Germany) was used as the standard, and the results are expressed as Trolox equivalents (µmol TE per 100 g).

##### Ferric Reducing Antioxidant Power

This assay is based on single electron transfer (SET) and measures the ability of an antioxidant to reduce ferric (Fe^III^) to ferrous (Fe^II^) ions (Benzie and Strain, 1996). The test protocol was described by Tienaho et al. (2021). In brief, a series of four dilutions of each sample, with three technical replicates, in a 96-well format were used in the assay. The samples were mixed with 20 mM FeCl_3_•6H_2_O (Sigma-Aldrich Chemie GmbH, Steinheim, Germany) and 10 mM 2,4,6-tris-(2-pyridyl)-s-triazine (TPTZ) (Sigma-Aldrich Chemie GmbH, Steinheim, Germany) in 300 mM acetate buffer (pH 3.6). The absorbance was measured at 594 nm with a microplate fluorescence reader (Varioskan Flash, Thermo Scientific) after the formation of the ferrous-tripyridyltriazine complex in the reaction mixture. FeSO_4_•7H_2_O (Sigma-Aldrich Chemie GmbH, Steinheim, Germany) was used as the standard and L(+)-ascorbic acid (150 and 800 µM) (VWR Chemicals) as the control. The results are expressed as µmol/L Fe(II) equivalents.

##### *The Hydrogen Peroxide (H*_*2*_ *O*_*2*_ *) Scavenging*

Activity was determined using a method modified from Hazra et al. (2008) and Jiang et al. (1990). The experimental setup has been described in detail by Tienaho et al. (2020). In brief, an aliquot of 2 mM H_2_O_2_ (Merck KGaA, Darmstadt, Germany) was added to the reaction mixture with the sample and a mixture containing 2.56 mM ammonium ferrous (II) sulfate (BDH Prolabo) in 0.25 mM H_2_SO_4_ (Merck KGaA) and 27.8 µM xylenol orange disodium salt (Sigma-Aldrich Chemie GmbH, Steinheim, Germany) in 4.4 mM sorbitol (D(-)-sorbitol, AppliChem GmbH). After 30 min of incubation, the absorbance of violet-colored ferric-xylenol orange complexes at 560 nm was measured. The assay measures the ability of the sample to scavenge H_2_O_2_ and prevent the oxidation of Fe(II) to Fe(III), which is indicated by the formation of the ferric-xylenol orange complex. The inhibition of oxidation is expressed as the inhibition (%) of the reaction, and the samples with 100% inhibition activity will remain yellowish. Sodium pyruvate (Sigma-Aldrich) was used as a reference compound.

##### Folin-Ciocalteu Assay

The Folin-Ciocalteu method (Singleton and Rossi, 1965; Singleton et al., 1999; Ainsworth and Gillespie, 2007) was used to analyze the total phenolic content, which is known to reflect antioxidant capacity. The test protocol was described by Tienaho et al. (2021). In brief, the samples were mixed with Folin-Ciocalteu reagent (Merck KGaA, Darmstadt, Germany) and 20% Na_2_CO_3_ (Merck KGaA, Darmstadt, Germany), and absorbance was measured at 750 nm with gallic acid (Sigma-Aldrich Chemie GmbH, Steinheim, Germany) as a reference compound. The results are expressed as gallic acid equivalents per gram (GAE/g).

#### 2.4.2 Antibacterial Properties

Two constitutively light-emitting bacterial biosensor strains, *E. coli* K12 + pcGLS11 and *S. aureus* RN4220 + pAT19, described by Vesterlund et al. (2004), were used in the present study. Bacterial cultivation and stocks were grown as previously described (Välimaa et al., 2020). Briefly, the bacteria were stored at –80°C and cultivated for approximately 16 h at 30°C (*E. coli*) and 37°C (*S. aureus*) on lysogeny agar (LA) plates (tryptone 10 g/L; yeast extract 5 g/L; NaCl 10 g/L; and agar 15 g/L). The LA plates were supplemented with 10% (v/v) sterile filtered phosphate buffer (PB) (1 M, pH 7.0) and 100 μg/ml of ampicillin (*E. coli*) or 5 μg/ml erythromycin (*S. aureus*). Biosensor stocks were prepared by inoculating a single colony of bacteria in lysogeny broth supplemented with 10% (v/v) of sterile filtered PB 1 M (pH 7.0), 100 μg/ml ampicillin (*E. coli*), or 5 μg/ml erythromycin (*S. aureus*). Stocks were cultivated for approximately 16 h at 300 rpm shaking at 30°C (*E. coli*) and 37°C (*S. aureus*). Extractions of all the willow clones in Table 1 were diluted in double-distilled water to achieve a 5% v/v concentration per microplate well. Ethanol 35% per microplate well was used as a positive control, and double-distilled water was used as a negative control. The reference substances, picein, triandrin, salicin, salicylic acid, and Salixin Organic Powder were diluted in double-distilled water to achieve concentrations of 250 μg/ml and 125 μg/ml per microplate well, whereas Salixin Organic Extract was used in 2.5% and 1.25% v/v per microplate well. Aliquots of 50 µl of samples and controls were pipetted in triplicate into opaque white polystyrene microplates, and 50 µl of bacterial culture was pipetted into the same wells. The luminescence was then measured using a Varioskan Flash Multilabel device (Thermo Scientific) once every 5 min for 95 min at room temperature, and the plate was briefly shaken before every measurement. The results are expressed as relative light units (RLUs) drawn at a time point of 40 min of measurement. Error bars represent the standard deviations between the sample triplicates.

#### 2.4.3 Antiviral Activity

Enterovirus coxsackievirus A9 (CVA9; Griggs strain, ATCC) was used to assess the antiviral efficacy of the *Salix* spp. clone extracts. CVA9 was produced and purified using a sucrose gradient, as previously described (Ruokolainen et al., 2019). Pretreatment of CVA9 [2 × 10^6^ plaque-forming unit (PFU) per ml] was performed with different amounts of *Salix* spp. extract (0.1%, 1%, and 10% v/v). After 1 h incubation at 37°C, the virus-*Salix* spp. mix was diluted 10-fold in 10% Dulbecco’s Modified Eagle Medium (DMEM). The mixture was added to the lung carcinoma cell line A549 (ATCC) containing 96-well plates with 12,000 cells/well density, plated on the previous day. After 48 h, the wells were washed twice with phosphate-buffered saline (PBS) and stained with crystal violet solution (0.03% crystal violet, 2% ethanol, and 36.5% formaldehyde) for 10 min, as described previously (Ruokolainen et al., 2019). The color was left in the wells because the remaining surviving cells were dissolved in a homogenization solution (0.8979 g of sodium citrate and 1 N HCl in 47.5% ethanol). The absorbance was measured spectrophotometrically at 570 nm using a PerkinElmer VICTOR^TM^ X4 multilabel reader. Cytotoxicity of the *Salix* spp. preparations was evaluated using the crystal violet solution mentioned above (Ruokolainen et al., 2019).

#### 2.4.4 Antifungal Activity

Quantitative suspension tests for the evaluation of basic fungicidal and basic yeasticidal activity of the *Salix* spp. samples were performed according to DIN EN 1275 (2006) and European Standard (2006). The fungicidal activity was evaluated using *A. brasiliensis* (strain ATCC 16404) and the yeasticidal activity using *C. albicans* (ATCC 10231) as a test organism. The microorganism suspension used ranged between 1.5×10^7^ colony forming units (CFU)/ml to 5.0 × 10^7^ CFU/ml for *A. brasiliensis* and *C. albicans*. For both organisms, the choice of the test method was the dilution-neutralization method using Tween80 + lecithin (30 g/L polysorbate 80 and lecithin 3 g/L) as a neutralizer. Sterile double-distilled water was used as the diluent during the test. The test concentrations for the samples Salixin Organic Extract 800NP, Salixin Organic Powder, and Klara (2-L scale sample P-16) were 10, 2.5, 1.25, 0.625, 0.3125, 0.156, 0.078, and 0.039 mg/ml. Unlike that described for EN 1275, in some cases, total test volume of 5 ml was used in the experiments instead of 10 ml. The contact time and test temperature were 15 min (±10 s) and 20°C (±1°C), respectively. In addition, the following higher test concentrations were evaluated for the samples: Klara P-16 100 mg/ml, Salixin Organic Powder 100 mg/ml, Salixin Organic Extract 100%, 50%, 25%, 10%, and 5% (v/v), and lyophilized Salixin Organic Extract 50% and 100%.

The number of viable spores was assessed on malt extract agar (four replicates). Plates were incubated for 42–48 h at 30°C, *A. brasiliensis* for a further 20–24 h, and viable spores were determined by counting the colonies (colony counts less than 300 CFU/plate). The reduction in viability is the ratio N/N0, where N0 is the number of CFU/ml in the fungal spore test suspension and N is the number of CFU/ml after the test procedure for the fungicidal activity of the product. The sample is fungicidal or yeasticidal if it produces at least a 10^4^ reduction in the number of viable vegetative yeast cells and mold spores under conditions defined by EN 1275 (2006).

### 2.5 Statistical Methods

Relationships among *Salix* spp. clone extract bioactivities were evaluated by Pearson’s correlation coefficients (in absolute values), and the differences between mean values were assessed using a two-tailed *t*-test with a significance level of 0.05 (*n* = 16 for ORAC, FRAP, and Folin-Ciocalteu tests; *n* = 17 for the *E. coli*, *S. aureus*, and Enterovirus test). Statistical differences between the grouped clones were determined by one-way ANOVA and Tukey post-hoc test, where results were determined to be statistically significantly different if *p*-values were below 0.05. All statistical analyses were performed using IBM SPSS Statistics (v. 26.0) (IBM, Armonk, NY, United States).

## 3 Results

### 3.1 Antioxidant Properties

**FIGURE 3:**
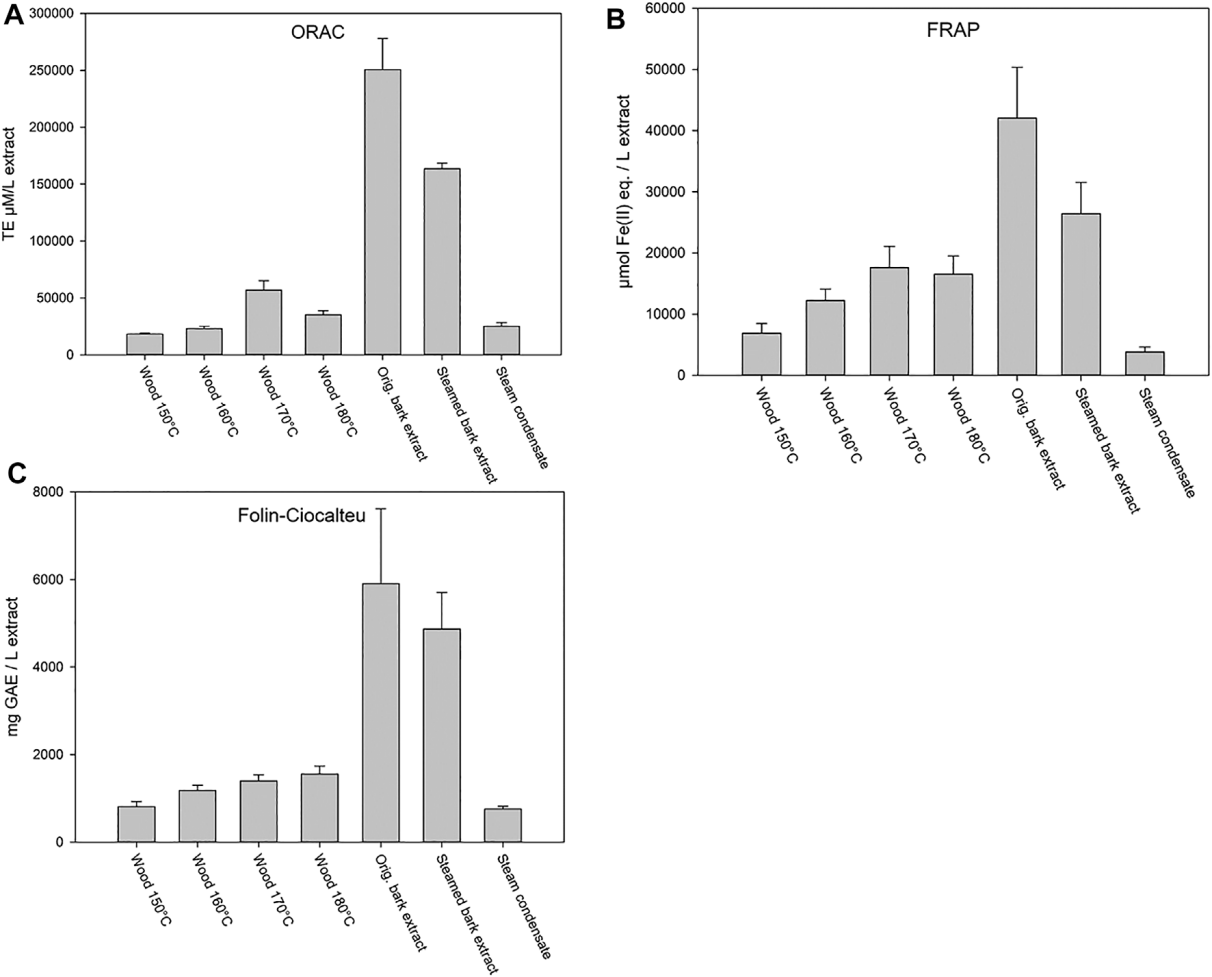
ORAC **(A)** and FRAP **(B)** activities, and total phenolic content (Folin-Ciocalteu) **(C)** of willow wood after debarking extracted at different temperatures (150, 160, 170, and 180°C) and bark extracts (original bark extract, steam-treated bark extract, and steam condensate). Error bars present the standard deviations of the sample triplicates in a microplate. ORAC test results are expressed as Trolox equivalents (TE), FRAP results are expressed as ferrous ion equivalents (Fe(II) eq.), and Folin-Ciocalteu test results are expressed as gallic acid equivalents (GAE).

The steam treatment results in Figure 3 show that the highest antioxidant potential and phenolic content (Folin-Ciocalteu) were found in the original willow bark ASE-350 extract. The steamed bark extract also showed elevated antioxidant activity, while the different hot water flow-through extracted wood samples at temperatures of 150–180°C, high in carbohydrates and hemicelluloses, showed much lower antioxidant potential. Reductions in both antioxidant activities (FRAP and ORAC) and total phenolic content were observed when compared to that of original bark extract (Figure 3), indicating that some phytochemicals are leached away. In addition, steam condensate showed some activity, which further supported the partial loss of compounds.

**FIGURE 4:**
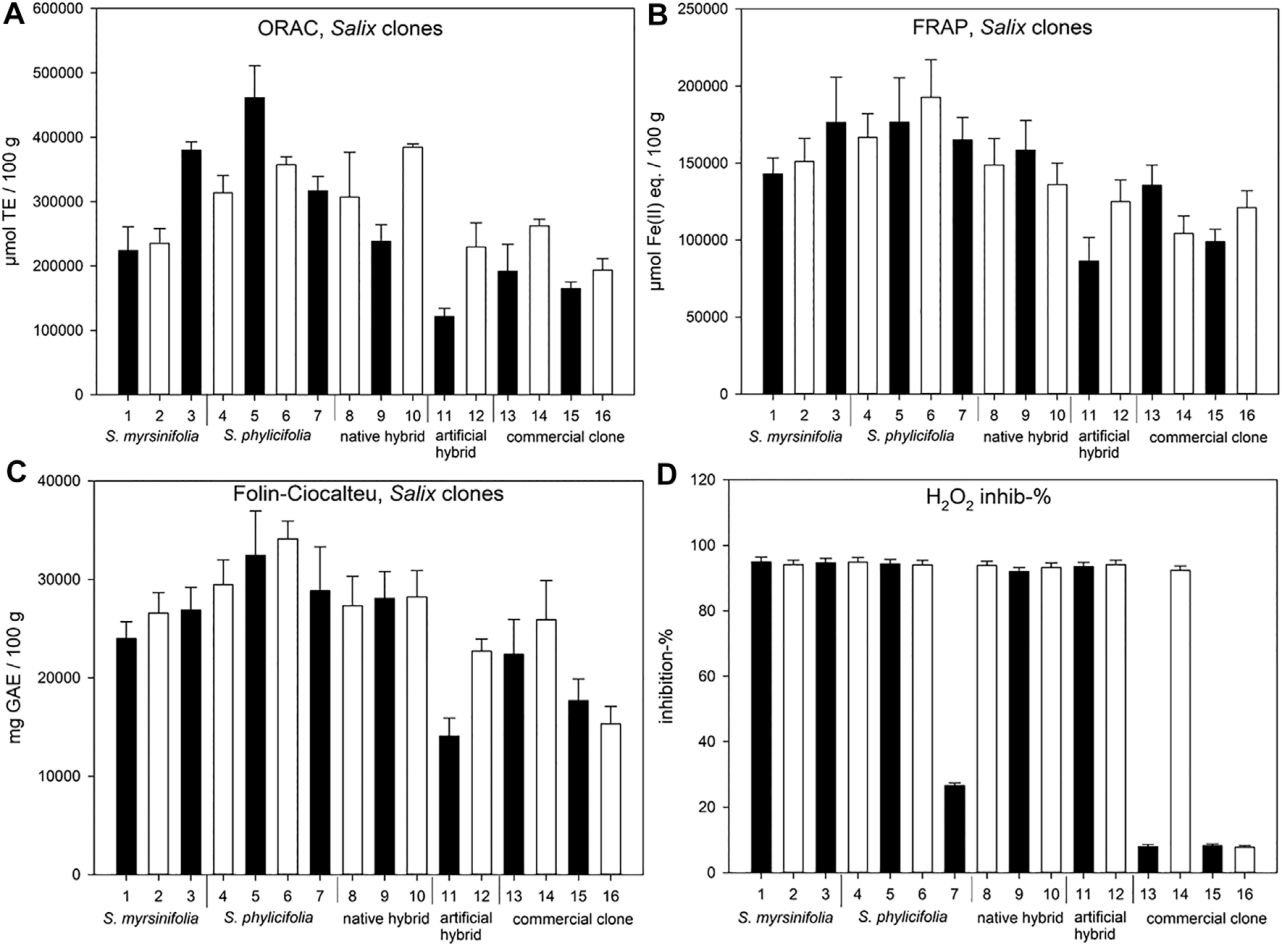
ORAC **(A)**, FRAP **(B)**, and H_2_O_2_ scavenging **(D)** activities, as well as total phenolic content (Folin-Ciocalteu) **(C)** of the *Salix* spp. clone extracts. Error bars present the standard deviations of the sample triplicates in a microplate. The clones are numbered 1–16 (see Table 1) and 13–16 are commercial clones. ORAC test results are expressed as Trolox equivalents (TE), FRAP results are expressed as ferrous ion equivalents (Fe(II) eq.), Folin-Ciocalteu test results are expressed as gallic acid equivalents (GAE), and hydrogen peroxide scavenging test results are expressed as the percentage of H_2_O_2_ inhibition.

The *Salix* spp. clone extract results (Figure 4) showed that the highest ORAC activity (µmol TE/100 g) was obtained with clone number 5, while clones 3, 6, and 10 also showed high ORAC activity. Clone extract 6 showed the highest FRAP efficiency, while clone extracts 3, 4, and 5 also showed high activity. All clone extracts except 7, 13, 15, and 16 showed high activity in the hydrogen peroxide scavenging test. The highest total phenolic content was found in the clone extract 6. Commercial clone extracts 13, 15, and 16 showed very low hydrogen peroxide scavenging activity (Figure 4D).

**FIGURE 5:**
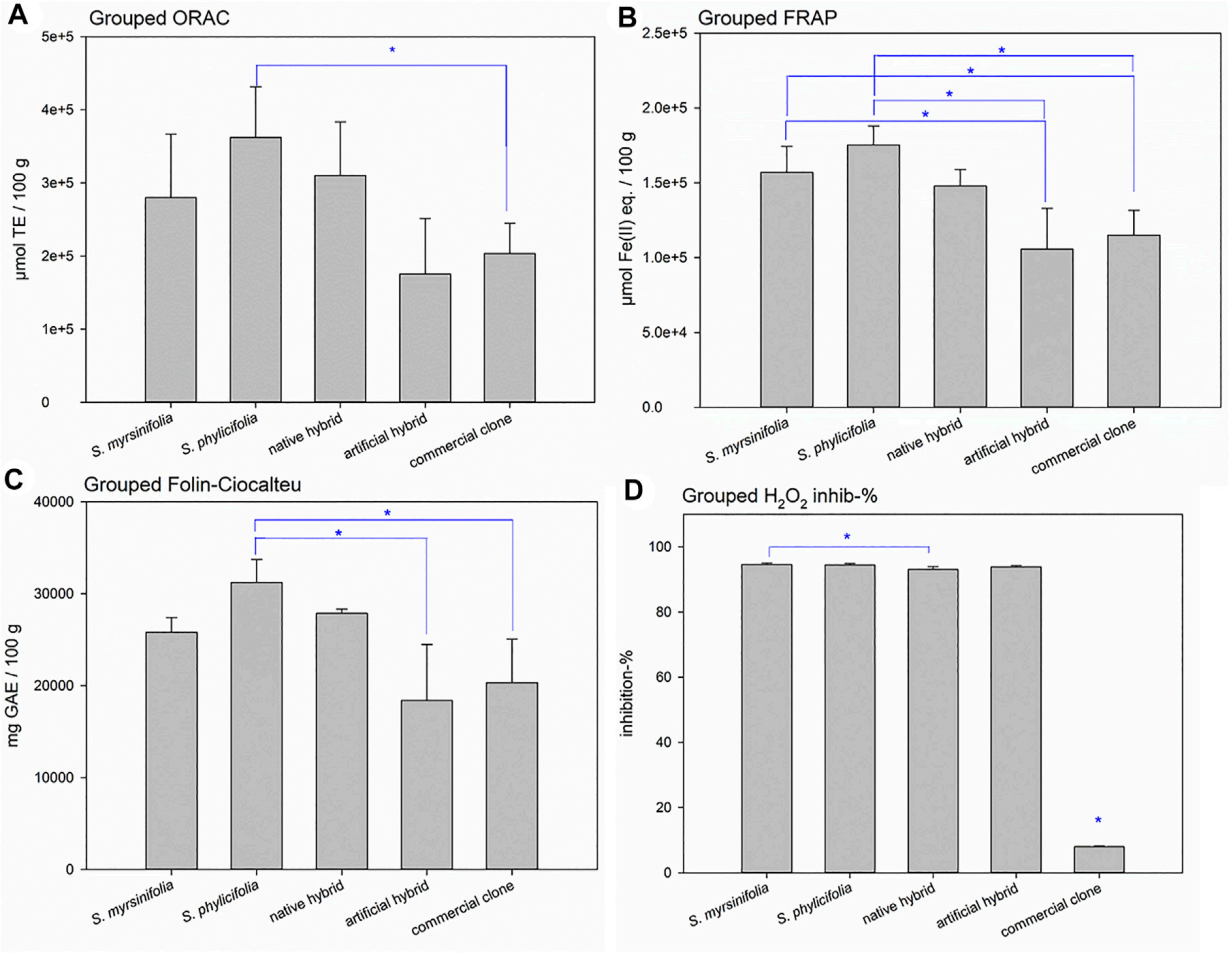
Grouped ORAC **(A)**, FRAP **(B)**, and H_2_O_2_ scavenging **(D)** activities, as well as total phenolic content (Folin-Ciocalteu) **(C)** of the *Salix* spp. clone extracts. Error bars show the standard deviation between the grouped clones. Significant differences are indicated with a blue asterisk. ORAC test results are expressed as Trolox equivalents (TE), FRAP results are expressed as ferrous ion equivalents (Fe(II) eq.), Folin-Ciocalteu test results are expressed as gallic acid equivalents (GAE), and hydrogen peroxide scavenging test results are expressed as the percentage of H_2_O_2_ inhibition.

When grouping the clone extract antioxidant results into *S. myrsinifolia* (clones 1–3), *S. phylicifolia* (4–7), native hybrid (8–10), artificial hybrid (11–12), and commercial clones (13–16), there was a significant difference between the groups as determined by one-way ANOVA [ORAC: *F*(4,11) = 4.012, *p* = 0.030; FRAP: *F*(4,11) = 10.102, *p* = 0.001; Folin-Ciocalteu: *F*(4,11) = 7.552, *p* = 0.004; and H_2_O_2_ scavenging with clones 7 and 14 emitted: *F*(4,9) = 14065.006, *p* < 0.001]. A Tukey post-hoc test revealed that *S. phylicifolia* clone extracts showed significantly higher activity in the ORAC test than the commercial clone extracts (*p* = 0.045) (Figure 5A), and significantly higher FRAP activity than the artificial hybrid extracts (*p* = 0.003) and commercial clone extracts (*p* = 0.002) (Figure 5B). In addition, *S. myrsinifolia* clone extracts showed significantly higher FRAP activity than the artificial hybrid extracts (*p* = 0.036) and commercial clone extracts (*p* = 0.041) (Figure 5B). *S. phylicifolia* clone extracts also showed statistically significantly higher total phenolic compound capacity in the Folin-Ciocalteu test than artificial hybrid extracts (*p* = 0.009) and commercial clone extracts (*p* = 0.006). In the H_2_O_2_ scavenging test, clone 7 gave inconsistently lower results than the other *S. phylicifolia* clone extracts, and clone 14 gave inconsistently high results when compared to the other commercial clone extracts. In dioecious plant species such as *Salix* spp., gender can be the reason for clonal differences in growth and wood quality, as shown by Hou et al. (2017). In this case, gender may not explain the differences in the activity, because all of the commercial clones included, also clone 14, were known to be female. However, when the outlier clones 7 and 14 were removed from the groups, *S. myrsinifolia* (*p* < 0.001), *S. phylicifolia* (*p* < 0.001), native hybrid (*p* < 0.001), and artificial hybrid (*p* < 0.001) clone extracts showed significantly higher antioxidant activity in the H_2_O_2_ scavenging test than commercial clone extracts (Figure 5D). In addition, *S. myrsinifolia* clone extracts showed significantly higher activity (*p* = 0.049) than native clone extracts (Figure 5D).

### 3.2 Biosensor Analysis and Antibacterial Activity

**FIGURE 6:**
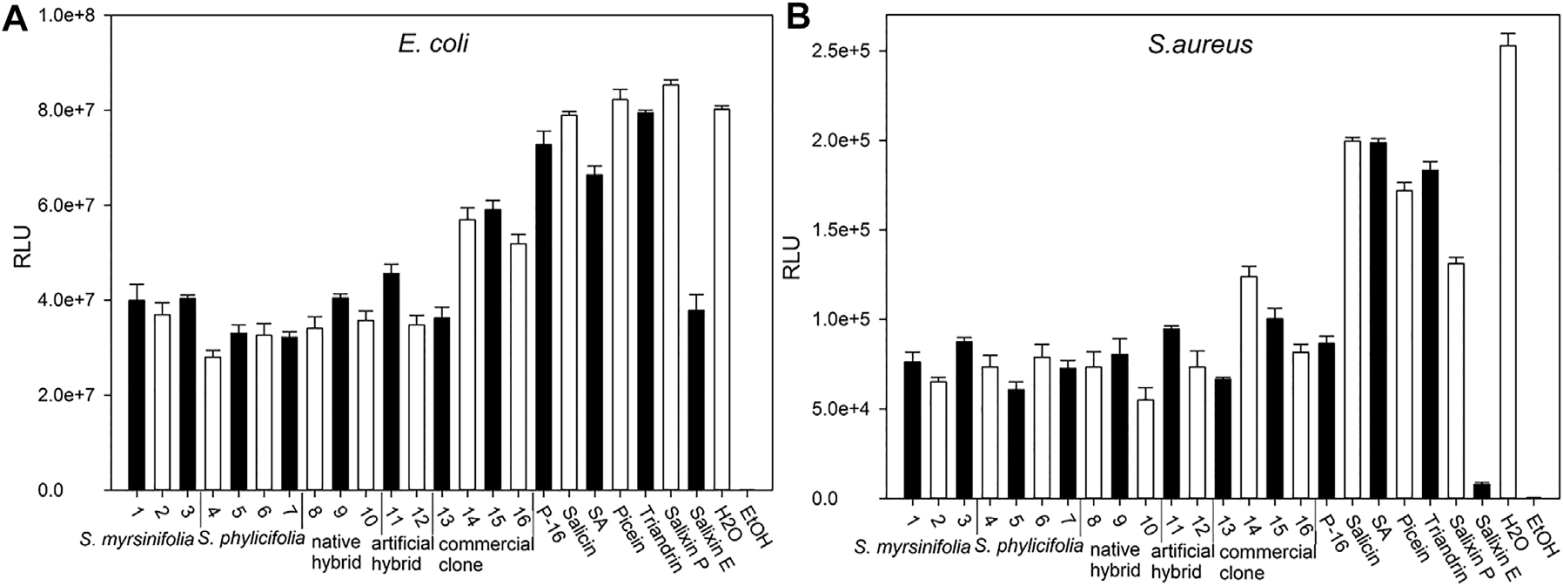
The bacterial biosensor results. Efficacy against *E. coli* **(A)** and *S. aureus* **(B)** after 40 min incubation time. The *Salix* spp. clones (Table 1) of 5% v/v concentration per a microplate well are indicated with numbers 1–16. P-16 = 2-L scale clone 16; commercial substances Salixin P = Salixin Organic Powder 48^TM^ (250 μg/ml) and Salixin E = Salixin Organic Extract 800NP^TM^ (1.25% v/v); SA = salicylic acid (250 μg/ml). Results obtained for salicin, triandrin, and picein are also shown at the concentration of 250 μg/ml per microplate well. Error bars indicate the standard deviation between the sample triplicates in the microplate. Lower RLU values indicate stronger antibacterial activity.

The antibacterial activity was evaluated using recombinant constituently bioluminescent strains of the leading bacterial pathogens of healthcare-associated infections and bacteremia: *E. coli* and *S. aureus* (Poolman and Anderson, 2018). The results are shown in Figure 6. Tienaho et al. (2015) showed that when using whole-cell bacterial biosensors, the empirical conditions could take from 10 to 15 min of incubation to stabilize. As shown in Figure 6, all the *Salix* spp. clone extracts had antibacterial activity, as evidenced by the lower RLU values than the negative control (water) with both bacterial strains after 10 min of incubation. The lower RLU values imply an inhibition of bacterial luminescence production and, thus, interference with bacterial metabolism. The highest inhibition effect after 40 min incubation was achieved by clone extract 4 in *E. coli* and clone extract 10 in *S. aureus*. However, the differences between the clones were small, with the exceptions of commercial clone extracts 14–16 and clone extract number 14, which had a somewhat lower effect on *E. coli* and *S. aureus*, respectively. For both bacterial strains, all the clone extracts exhibited stronger antibacterial activity than the control substances, salicin, salicylic acid, picein, and triandrin. The lowest inhibition seemed to be achieved with the 2-L stirring reactor extracted clone 16 (P-16); however, it still had lower luminescence production (in RLU) than the water control. The commercial reference substances showed lower antibacterial activity against both bacterial strains, except for the Salixin Organic Extract, which was equally active as the willow clone extracts with *E. coli* and showed almost as high antibacterial activity as ethanol (35%) with *S. aureus*. However, Salixin Organic Extract was considerably darker than the other samples, and this could have an effect on light reduction. The disadvantages of this method have been minimized by using small concentrations of dark-colored samples to reduce the effect of color and by repeating the measurement three times to ensure comparability between measurements (Tienaho, 2020).

**FIGURE 7:**
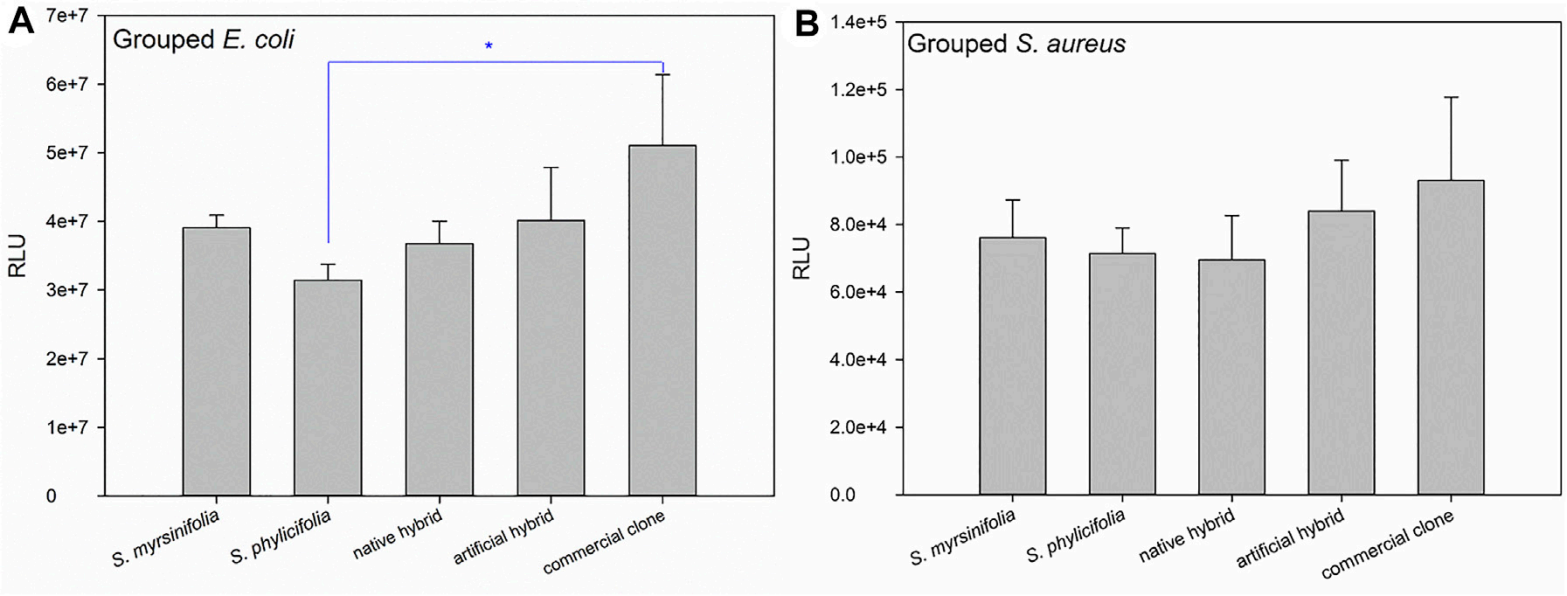
The grouped antibacterial results against *E. coli* **(A)** and *S. aureus* **(B)**. Error bars show the standard deviation between the grouped clones. Significant differences are indicated with a blue asterisk.

When grouping the clone extract results into *S. myrsinifolia* (clones 1–3), *S. phylicifolia* (4–7), native hybrid (8–10), artificial hybrid (11–12), and commercial clones (13–16), there was a significant difference between the groups as determined by one-way ANOVA in the *E. coli* biosensor results [*F*(4,9) = 5.266, *p* = 0.013], whereas the differences in the *S. aureus* results were statistically insignificant (Figure 7B). A Tukey post-hoc test revealed that *S. phylicifolia* clone extracts had significantly higher antibacterial activity against *E. coli* than the commercial clone extracts (*p* = 0.007) (Figure 7A). Other results were statistically similar.

### 3.3 Antiviral Activity

**FIGURE 8:**
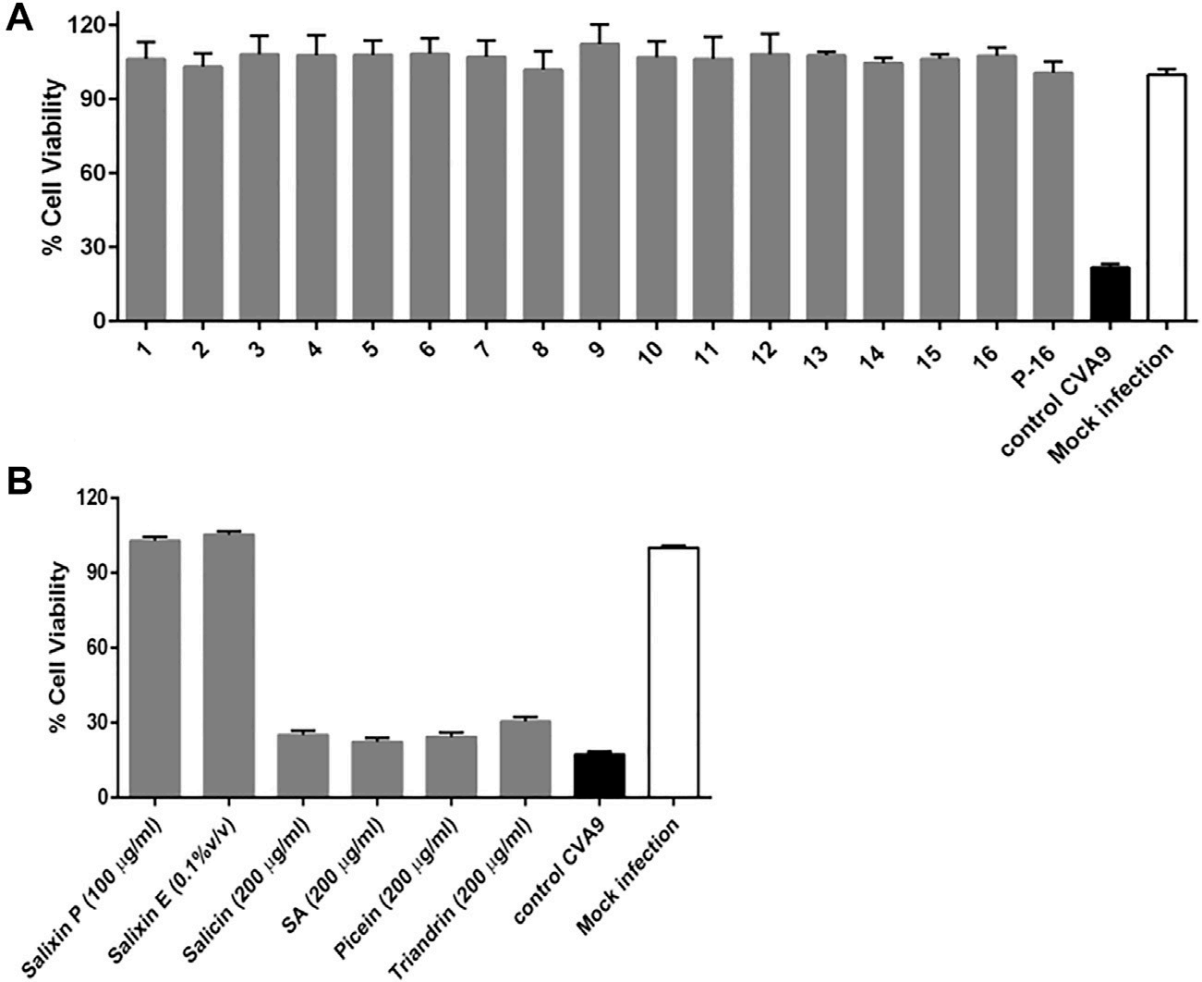
Testing the antiviral activity of **(A)** *Salix* spp. extracts (0.1% v/v) and **(B)** reference compounds (salicin, salicylic acid, picein, and triandrin) and Salixin Organic Powder and Extract against CVA9 using CPE inhibition assay. Virus control and test samples are normalized against a mock infection. The results are mean of two independent experiments with *n* = 3. The average values + standard errors of mean (SEM) are shown. P-16 = 2-L scale clone 16; Salixin P = Salixin Organic Powder 48^TM^; Salixin E = Salixin Organic Extract 800NP^TM^; SA = salicylic acid.

The antiviral activity was evaluated using the highly stable non-enveloped enterovirus CVA9. There are more than 100 serotypes of enteroviruses with very similar structures and functions (Marjomäki et al., 2015). To date, there are no clinically approved antivirals against enteroviruses or any non-toxic natural compounds that can safely disinfect enteroviruses or other non-enveloped viruses from surfaces. Here, the tested *Salix* spp. preparations proved to be very efficient against CVA9 (Figure 8A). Pre-incubation of the virus with *Salix* spp. extracts fully rescued the A549 cells from infection, proving that the extracts directly bound to the capsid. *Salix* spp. samples did not show any cytotoxicity (data not shown). We also tested Salixin Organic Extract and Powder along with the reference compounds (salicin, salicylic acid, picein, and triandrin). Similar to *Salix* spp. samples, Salixin Organic Powder and Extract also showed antiviral activity and protected the cells against CVA9 infection, whereas none of the reference compounds were effective in stopping the viral infection (Figure 8B).

**FIGURE 9:**
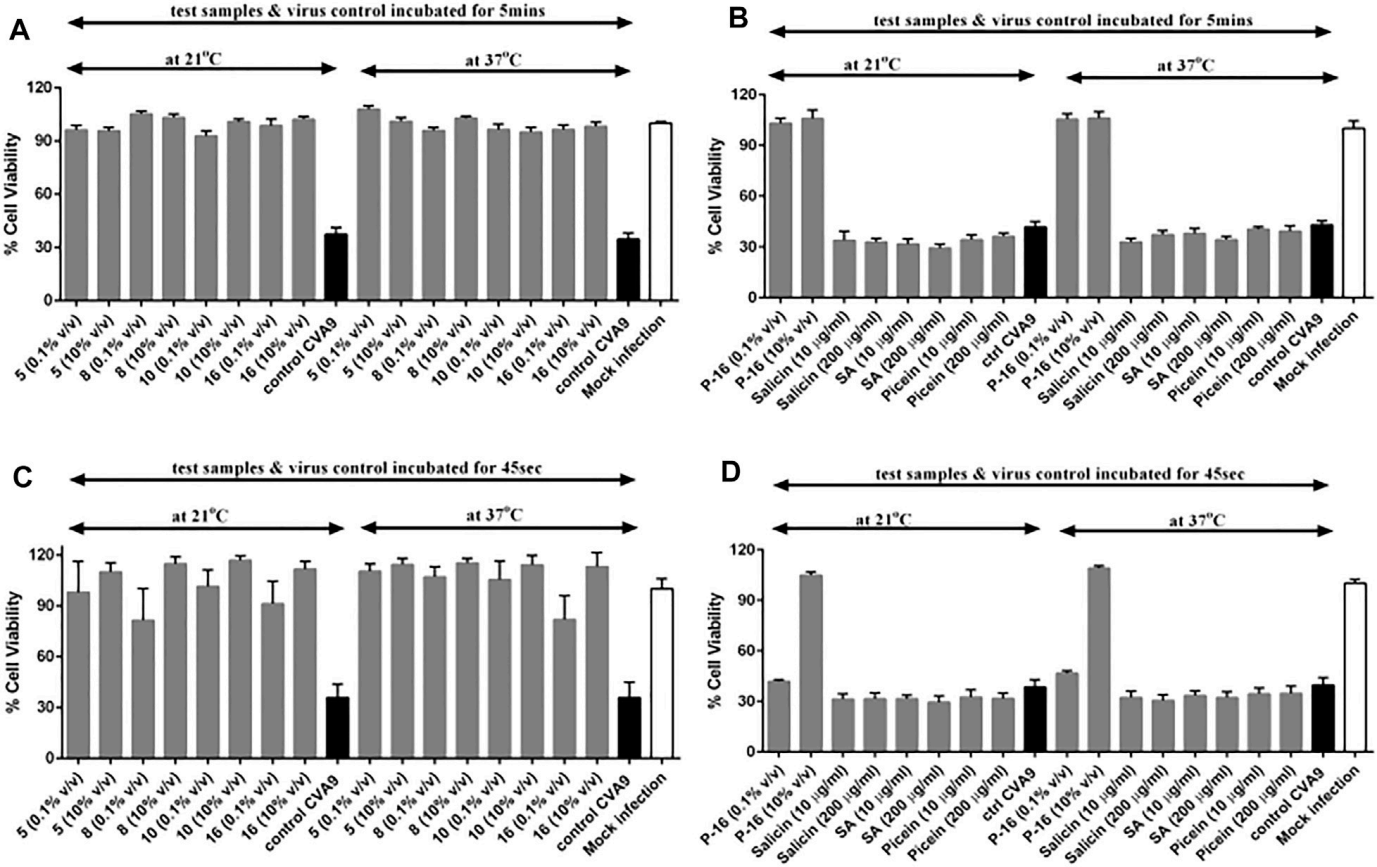
Effect of time and temperature on the antiviral activity of selected *Salix* spp. extracts and reference compounds using CPE inhibition assay. *Salix* spp.-virus mix was incubated at 37°C and 21°C for **(A, B)** 5 min and **(C, D)** 45 s. Virus control and test samples are normalized against a mock infection. The results are mean of two independent experiments, with *n* = 3. The average values + standard errors of mean (SEM) are shown. P-16 = 2-L scale clone 16; SA = salicylic acid.

To study the impact of time and temperature on the antiviral activity of *Salix* spp. extracts in further detail, we incubated selected *Salix* spp. samples with CVA9 for a shorter time interval (5 min) and at lower temperatures (21°C vs. 37°C). The selected *Salix* spp. extracts retained their antiviral efficacy even at room temperature (21 °C) and were able to stop CVA9 infection within 5 min of incubation at both temperatures tested (Figures 9A,B). None of the reference compounds tested showed antiviral activity (Figure 9B). We tested an even shorter incubation time (45 s) to further determine the efficacy of the *Salix* spp. extracts. Interestingly, this short pre-treatment with the *Salix* spp. extracts was sufficient for the samples to exert their antiviral efficacy and protect the cells from CVA9 infection (Figures 9C,D). However, the 2-L stirring reactor extracted clone 16, sample P-16, did not show antiviral activity at lower concentrations (0.1% v/v) when incubated with CVA9 for 45 s at 21°C or 37°C. Nevertheless, it completely protected the cells when tested at higher concentrations (10% v/v) (Figure 9D). These results indicate that the *Salix* spp. extracts can effectively block CVA9 infection within a few seconds of interacting with the virus at room temperature by acting directly on the virus capsid.

When grouping the clone extract results from Figure 8 into *S. myrsinifolia* (clones 1–3), *S. phylicifolia* (4–7), native hybrid (8–10), artificial hybrid (11–12), and commercial clones (13–16), there were no statistical differences between the groups as determined by one-way ANOVA [*F*(4,11) = 0.276, *p* = 0.887].

### 3.4 Antifungal Activity

The tested concentrations of Salix extract Klara (the 2-L scale sample) and Salixin Organic Extract and Powder were not effective against fungi (*A. brasiliensis*) and yeast (*C. albicans*). A reduction in viability higher than 4 log units, as required by the EN 1275 norms to qualify the product with fungicidal or yeasticidal efficiency, was not detected. However, higher concentrations of the commercial Salixin Organic Extract (50 and 100% v/v) showed minor inhibition against both *C. albicans* and *A. brasiliensis*.

## 4 Discussion

In the present study, we introduced a steam debarking method, which loosens the bark and allows its efficient removal; therefore, this method has the potential to decrease the costs of willow debarking. However, our results showed that some antioxidant activity was lost in the steam-treated bark samples. The original bark extract had higher polyphenol content and antioxidant capacity than the steam-treated bark extract, indicating that some phytochemicals were leached away in the process. One of the major constituents of various biomasses is lignin, which has been found to possess anti-inflammatory and antioxidant activities, and it has been found to degrade at high temperatures (Gu et al., 2021; Wang et al., 2021; Zheng et al., 2021). However, the steam-treatment temperatures did not rise above 180°C, and the decomposition of lignin, which is rather slow, has been considered to start at temperatures over 180°C (Nassar and MacKay, 1984; Brebu and Vasile, 2010). In the hydrothermal steam treatment of *Populus deltoides* (W. Bartram ex Marshall, Salicaceae), lignin content was only slightly decreased (Bobleter, 1994). Therefore, it is more likely that the degradation of other extractives, such as salicinoid or polyphenol structures, caused the decrease in the antioxidant capacity after steam treatment, especially if salicinoid structures were unstable (Julkunen-Tiitto and Sorsa, 2001; Ruuhola and Julkunen-Tiitto, 2003). Additionally, a substantial degradation of acetyl groups in hemicellulose can be expected at the temperature of 180°C (Steinbach et al., 2017). Antioxidant activity has been reported at least for xylo-oligosaccharide (hemicellulose model compounds) (Wu et al., 2019) and corn bran hemicellulose fragments (Ohta et al., 1994). However, without further structural characterization, any certainty is difficult to accomplish, and this poses a great opportunity for subsequential experimental studies.

All the tested concentrations of *Salix* spp. extracts and the commercial Salixin Organic Extract and Powder were effective against the non-enveloped CVA9. Enteroviruses such as CVA9 cause many acute and chronic infections on a yearly basis (Marjomäki et al., 2015). To the best of our knowledge, no previous studies have investigated the effectiveness of willow extracts directly against non-enveloped enteroviruses. However, effectiveness of natural compounds found in *Salix* spp. has been established for viruses sealed with lipid envelopes. For example, a review by Grienke et al. (2012) covered several studies performed between 2000 and 2011 on natural products specifically targeting the viral surface protein neuraminidase of influenza virus. They showed that the majority of the active natural products with the desired activity belonged to flavonoids, while (oligo)stilbenes, coumarins, and diarylheptanoids exhibited lower activity (Grienke et al., 2012). Liu et al. (2008) found that the activity for flavonoids was highest in aurones followed by flavon(ol)es, isoflavones, and flavanon(ol)es and flavan(ol)es, in this order, and that the C4′-OH, C7-OH, C4-O double bond, and C2-C3 double bond functionalities were essential for the inhibitory activity of these compounds.

Here, we showed that salicin, picein, salicylic acid, and triandrin were not responsible for the antibacterial and antiviral activities detected, at least not alone. *Salix* spp. clone extracts are highly antibacterial even at low concentrations and show similar luminescence light reduction as the bark extracts from Norway spruce [*Picea abies* (L.) H. Karst] found in Välimaa et al. (2020). Salicinoids are not present in spruce, and they are unstable and prone to decomposition especially with higher molecular masses (Julkunen-Tiitto and Sorsa, 2001; Ruuhola and Julkunen-Tiitto, 2003). This could indicate that other secondary metabolites, such as tannins, could affect bactericidal efficacy. In addition, none of the reference compounds showed antiviral activity against non-enveloped CVA9. This indicated that the presence of bioactive compounds other than the reference compounds tested here (e.g., picein and triandrin) is responsible for the antiviral activity of the *Salix* spp. extracts (cf. Dou et al., 2021). One interesting option for future studies is the tannins, which are likely to be found in the willow bark water extracts and have recently been associated with antiviral efficacies (e.g., Vilhelmova-Ilieva et al., 2019; Fraga-Corral et al., 2021). Contradictory results and hypotheses exist on whether polyphenols influence the overall bioactive efficacy of willow extracts (Khayyal et al., 2005; Nahrstedt et al., 2007; Antoniadou et al., 2021). Therefore, more research is needed to achieve any certainty regarding their effects on the bioactivities of willow extracts. However, extracts containing a mixture of willow compounds could also have synergistic effects. Similar observations were made by Shara and Stohs (2015), who concluded that the typical effective dosage of aspirin is twice that of salicin needed in willow bark extract, probably because of the presence of beneficial polyphenols and flavonoids in the bark extract.

The results obtained in the present study demonstrate the excellent antiviral, antioxidant, and antibacterial effects of *Salix* spp. bark extracts. Pearson’s linear correlation coefficient absolute values (Table 2) indicated either a strong or moderate relationship between *E. coli* and *S. aureus* antibacterial test results (60%) for the *Salix* clones, both bacteria with enterovirus results (*E. coli* vs. entero: 67%; *S. aureus* vs. entero: 28%), ORAC and FRAP values (76%), ORAC and total phenol content (84%), FRAP and total phenol content (85%), and *E. coli* and FRAP test results (75%). A strong relationship was obtained between *E. coli* and ORAC test values (61%), *E. coli* and total phenol results (67%)*, S. aureus* and ORAC values (41%), and *S. aureus* and FRAP values (53%). A moderate relationship was obtained for *S. aureus* and total phenol content (38%) and for enterovirus results and FRAP values (47%) as well as enterovirus and total phenol content results (45%), and enterovirus and ORAC results (36%).

**TABLE 2:** Pearson’s correlation coefficients for the different bioactivity tests. Values 0.70–0.99 indicate a very strong relationship, values 0.40–0.69 indicate a strong relationship, values 0.30–0.39 indicate a mediocre relationship, values 0.20–0.29 indicate a weak relationship, and values 0.01–0.19 indicate no or negligible relationship. The upper part of the matrix indicates the *p*-values for the data determined by a two-tailed *t*-test (*n* = 16 for ORAC, FRAP, and Phenols tests, *n* = 17 for the *E. coli*, *S. aureus*, and Enterovirus test). Most of the *p*-values are below 0.05, which indicates significant differences.

	ORAC	*E. coli*	FRAP	Phenols	*S. aureus*	Enterovirus
ORAC	**1**	<0.05	<0.001	<0.001	0.113	0.167
*E. coli*	0.61	**1**	<0.001	<0.05	<0.05	<0.05
FRAP	0.76	0.75	**1**	<0.001	<0.05	0.065
Phenols	0.84	0.67	0.85	**1**	0.146	<0.01
*S. aureus*	0.41	0.60	0.53	0.38	**1**	0.28
Enterovirus	0.36	0.67	0.47	0.45	0.28	**1**

Fungal infections and fungal resistance to currently available antifungal drugs are increasing globally. Fungal infections, as well as their prevention and treatment, also remain largely understudied compared to other infectious diseases (Brown et al., 2012). Novel and safe antifungal drugs and agents are needed for currently less common fungi, such as the recently reported Zygomycetes causing rare and life-threatening mucormycosis infection in patients with COVID-19 (do Monte Junior et al., 2020). For long, Amphotericin B was the only antifungal medication available, and only during the past three decades has a wider spectrum of antifungal agents (e.g., triazoles and echninocandin antifungals) become available (Spanakis et al., 2006). Promising antifungal compounds, including phenolic compounds, have been found in plants (Arif et al., 2008). To the best of our knowledge, no previous studies have investigated the efficacy of willow extracts against fungi and yeasts. Unfortunately, the *Salix* spp. bark extracts tested here showed no antifungal or yeasticidal activity. The only sample with putative mild activity was the commercial Salixin Organic Extract, which showed a minor inhibition of both *C. albicans* and *A. brasiliensis* when considerably concentrated.

The bioactivities of the domestic (1–12) and commercial clone extracts (13–16) were surprisingly similar despite the differences in harvesting time, age, and growing conditions of these two groups. However, the grouped commercial clone extracts showed significantly lower antioxidant (in ORAC and FRAP) and antimicrobial (*E. coli*) activities, as well as lower total phenolic capacity (Folin-Ciocalteu), than *S. phylicifolia*. In the H_2_O_2_ scavenging tests, the commercial clones showed significantly lower activities than all the other groups, and the FRAP test evidenced lower activity than that of *S. myrsinifolia*. The native clones had already reached the stage of vegetative bud burst, whereas the commercial clones had no visible signs of vegetative growth, indicating that they were in a state of dormancy (Saska and Kuzovkina 2010). According to Förster et al. (2008), the number of secondary metabolites in the bark of willow clones decreased during the vegetative season from March to July. The domestic clones were 1 year younger than the commercial clones. Nissinen et al. (2018) found only a minor decrease in the stem phenolic concentrations of *S. myrsinifolia* during a 7-year study period. Instead, Tahvanainen et al. (1985a) showed a significantly higher concentration of phenolic glycosides in juvenile willow twigs than in mature twigs. In the present study, the domestic clones grew in small polystyrene containers and restricted conditions, which could have resulted in higher amounts of polyphenolic defense and resistance compounds. According to Paunonen et al. (2009), polythene mulching and fertilization increased the yield of foliar salicylates because of the enhanced leaf biomass, but not the salicylate concentration in *S. myrsinifolia* clones. Furthermore, they noted that the yields and concentrations of leaf phenolics seemed to be more influenced by the clone than by the cultivation method. According to Glynn et al. (2004), drought treatment did not affect the foliar phenolic concentrations of willow genotypes.

Small differences were observed in the antibacterial tests, where commercial clones 14–16 seemed to have a somewhat lower effect in *E. coli* and commercial clone 14 seemed to have a lower effect in *S. aureus*. The commercial clones 13, 15, and 16 also showed almost no H_2_O_2_ scavenging activity in the antioxidant tests. The total phenol content and the composition of phenolic compounds vary widely among willow species, and the composition of leaf phenolic glycosides is species-specific (Julkunen-Tiitto, 1986). *S. myrsinifolia* (former *Salix nigricans* Sm.) clearly differed from other native species in terms of high phenolic glycoside content in leaves, mostly due to salicortin (Tahvanainen et al., 1985b), and so did the introduced species *S. dasyclados*. According to Julkunen-Tiitto (1986), *S. phylicifolia* leaves contained the highest total phenolic content among the 15 Salicaceae species studied, with more than 15% of dry weight, whereas *S. myrsinifolia* leaves had the lowest total phenolic content. In the present study, four clones (clones 4–7) with the highest total phenol content in the bark extracts were identified as *S. phylicifolia*. In contrast, *S. myrsinifolia* bark and leaves were characterized by higher amounts of phenolic glycosides such as salicylates (Julkunen-Tiitto, 1985, 1986), whereas the low content of phenolic glycosides was typical of *S. phylicifolia* and *S. viminalis* L. The latter is the main species in the ancestry of the commercial clones examined here, showing slightly lower antibacterial effects. Clonal variation exists within species; for example, Paunonen et al. (2009) showed highly variable responses of *S. myrsinifolia* clones to fertilization and mulching treatments, and thus to the yield of foliar phenolics, and they concluded that willow cultivation for the herbal industry should be based on correct clone selection.

Bark extracts of *S. myrsinifolia* × *phylicifolia* hybrids (clones 8–10) did not statistically differ from other native clones in terms of bioactivity, except in the H_2_O_2_ scavenging antioxidant test, when clones 7 and 14 were removed. There was also a statistically significant difference between the higher inhibition-% values of *S. myrsinifolia* clone extracts compared to native hybrid extract values (*p* = 0.049). According to Julkunen-Tiitto (1986), these native hybrids resemble *S. phylicifolia* on the basis of total phenolics in leaves, but *S. myrsinifolia* by their foliar glycosidic composition (Julkunen-Tiitto, 1986). In Hallgren et al. (2003), F1-hybrids of *S. caprea* L. and *S. repens* L. were intermediate between their parental species in terms of foliar secondary metabolites and herbivore resistance but increased hybridization decreased this resistance. In the present study, clones 11 and 12 were hybrids of three species from controlled crossings, i.e., female (*S. myrsinifolia* × *phylicifolia*) × male *S. dasyclados*. In particular, the bark extract of clone 11 showed a somewhat lower total phenolic content and weaker antibacterial activity than the bark extracts of native species. In fact, all commercial clones are also outcomes of multiple crossings between different willow species, with *S. viminalis* being the most used ancestor, and characterized by low content of phenolic glycosides (Julkunen-Tiitto, 1985, 1986). Thus, both of these reasons may explain the significantly lower antibacterial effects of the commercial clones. Another possible cause of the difference in the activities of artificial hybrids and commercial clones could be the difference in the bioactive polyphenol compound composition or quantity, which would be interesting to study in the future. Further interesting questions arise, whether the beneficial antioxidant and antimicrobial activities and total phenolic capacity of *S. phylicifolia* could be increased by intraspecific crossings, or whether they are inevitably decreased by repeated species hybridization when, for instance, breeding for higher yield.

This study verified the antimicrobial potential of the willow bark biomass extracts. Antimicrobial and antiviral protection are required for a variety of industrial applications. For example, it has been estimated that by the year 2027, the antimicrobial packaging market would globally reach the value of 13.28 billion pounds (VMR Verified Market Research, 2020). It is noteworthy that viruses such as noroviruses, which cause the nasty outbreaks of stomach infection, are non-enveloped viruses such as the enterovirus CVA9 studied here. Non-enveloped viruses are much more difficult to decontaminate than enveloped viruses because of the tight protein package around the virus genome instead of the lipid envelope, which is more vulnerable to breakage. In addition to noroviruses, enteroviruses transmit easily through surfaces and cause acute and chronic infections. Currently, there are no effective and safe antivirals that directly act on virus particles. *Salix* spp. extracts offer a safe and affordable solution to combat these stable viruses. Antiviral and antimicrobial compounds from renewable willow could also replace products that may not be readily biodegradable.

For obtaining valuable phenolic compounds from willow bark, one of the limiting factors has been laborious debarking. In the present study, we showed that steaming can be used for fast and efficient debarking, while only some antioxidant potential is lost in the process. Thus, this method has potential at industrial scale.

In the European Union, plantations of short rotation coppice (SRC) willows have been established in the past, primarily for energy use purposes. Willows could offer advantages over mainstream commercial conifers owing to their fast growth and high productivity. SRC willows may also provide environmental benefits in terms of carbon sequestration when grown on marginal land, such as abandoned agricultural land or peatland (Rytter et al., 2015). For well-managed willows growing at the underappreciated peatland, the annual yield can reach up to >12.3 oven dry ton (odt)/ha, which exceeds about 8%–30% of the yield obtained with the domestic natural forest species (i.e., birch and grey alder) on the same land (Hytönen and Saarsalmi, 2009). In Finland, bioenergy-targeted projects in the 1980s produced knowledge on the hybridization, cultivation, and management of SRC willows for energy use. High biomass yields are achievable if cultivation is based on well-adapted, selected clones, and biotic and abiotic damages are avoided (Volk et al., 2004; Verwijst et al., 2013). This knowledge is a valuable foundation for creating willow biorefinery approaches for high added value. The comprehensive and optimized utilization of willow lignocellulosic biomass will promote sustainability and carbon neutrality, but requires further research, for example, on the life-cycle assessment of the production and processing value chain (cf. Rasi et al., 2019).

In conclusion, the present study provides novel information on the antioxidant, antibacterial, and antiviral properties of polyphenolic bark extracts of SRC willows well adapted to the Finnish growing environment, by using scalable green extraction techniques. We found that all the tested concentrations of *Salix* spp. extracts were effective against the non-enveloped enterovirus CVA9 as well as *E. coli* and *S. aureus* strains. However, there seemed to be more variation between the clones in their antioxidant activities determined by ORAC, FRAP, and H_2_O_2_ scavenging abilities. No marked efficacy was detected in the antifungal or yeasticidal tests. When clone extracts were grouped, *S. phylicifolia* clones showed the most promising antioxidant and antibacterial activities with significant differences when compared to commercial and artificial clones. This can partly be caused by the effect of stronger hybridization; however, more studies are needed to examine the possible effects of compound composition and content of the extracts. We also showed that salicin, picein, salicylic acid, and triandrin are not responsible for the antibacterial and antiviral activities detected here, at least not alone. Instead, other compounds of interest, such as polyphenols, or synergistic effects between the compounds, are likely to cause the detected efficacies. We also demonstrated for the first time that steam debarking is a promising, less-laborious method for the efficient separation of bark from wood (harvested in the spring season), with only minor effects on the antioxidant properties of bark. This method promotes the cascade use of willow biomass, where the debarked wood can be used for other purposes. Our findings will potentially lead to scientific breakthroughs given that the studied crude extracts with promising mixtures of components are highly effective against the stable non-enveloped viruses that cause nasty acute and chronic infections. Further investigations and development of such antiviral solutions for enveloped viruses are topics for our next studies. Biochemicals obtained from tree bark biomass and side products of biorefinery approaches have potential for various applications (e.g., health promotion, cosmetics, pharmaceuticals, packaging, coatings, other functional materials, and surfaces). Willow, as a short-rotation coppice species with a fast growth rate and high yield on marginal lands, presents a promising alternative for the currently most common commercial tree species in Finland.

## Data Availability

The original contributions presented in the study are included in the article/Supplementary Material. Further inquiries can be directed to the corresponding author.
