# Supplementary material for: Salix spp. Bark Hot Water Extracts Show Antiviral, Antibacterial, and Antioxidant Activities—The Bioactive Properties of 16 Clones
Source: Front Bioeng Biotechnol. 2021 Dec 16;9:797939. doi: 10.3389/fbioe.2021.797939 (PMC8716786; doi:10.3389/fbioe.2021.797939)
Supplement: Supplementary file 1 [file Table1.DOCX]

| **Table A.1. Willow clones used in the steam debarking experiment.** | | |
| --- | --- | --- |
| **Sample 1, 6-year-old** |  | |
| **Clone number** | **Species** | **"Origin"** |
| 1012 | *Salix purpurea* | Jyväskylä, Viherlandia |
| 1105 | *Salix purpurea* 'Lambertiana' | Unknown |
| 1106 | *Salix purpurea* 'Leentjes' | Unknown |
| 1108 | *Salix purpurea* 'Procumbens' | Unknown |
| 1109 | *Salix purpurea* 'Uralensis' | Unknown |
|  |  | Unknown |
| **Sample 2, 12-year-old** |  | |
| **Clone number** | **Species** | **"Origin"** |
| 1023 | *Salix* sp*.* | Ruissalo, 84-0759, Herog |
| 1037 | *Salix daphnoides* subspecies (ssp.) *acutifolia* | Jyväskylä, Forssa |
| 1038 | *Salix daphnoides* ssp. *acutifolia* "Arkadia" | HY, Viikki arboretum |
| 1061 | *Salix daphnoides* ssp. *daphnoides* | Ohenoja, Kiiminki |
| 1063 | *Salix daphnoides* ssp. *acutifolia* | Helsinki, Meilahti arboretum |
| 1064 | *Salix daphnoides* ssp. *daphnoides* | Jyväskylä, Oulu |
| 1086 | *Salix purpurea* 'Helix Pyramidalis' | Puola 3181 |
| 1088 | *Salix* sp. | Piikkiö, Yltöinen |
