# Supplementary figures and images for: Salix spp. Bark Hot Water Extracts Show Antiviral, Antibacterial, and Antioxidant Activities—The Bioactive Properties of 16 Clones
Source: Front Bioeng Biotechnol. 2021 Dec 16;9:797939. doi: 10.3389/fbioe.2021.797939 (PMC8716786; doi:10.3389/fbioe.2021.797939)

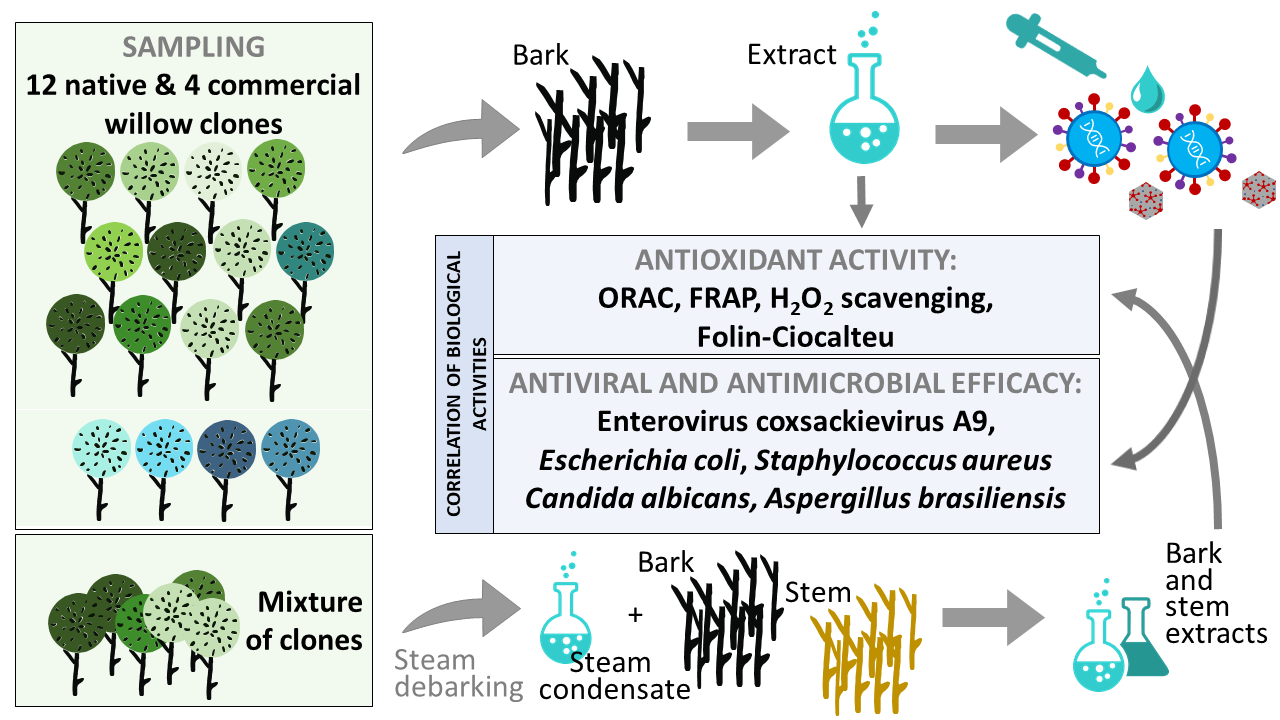

Supplement: Supplementary file 2 [file Image1.TIF]
